# Nonlinear Properties of Ge-rich Si_1−x_Ge_x_ Materials with Different Ge Concentrations

**DOI:** 10.1038/s41598-017-15266-z

**Published:** 2017-11-07

**Authors:** Samuel Serna, Vladyslav Vakarin, Joan-Manel Ramirez, Jacopo Frigerio, Andrea Ballabio, Xavier Le Roux, Laurent Vivien, Giovanni Isella, Eric Cassan, Nicolas Dubreuil, Delphine Marris-Morini

**Affiliations:** 1 Centre de Nanosciences et de Nanotechnologies, CNRS, Univ. Paris-Sud, Université Paris-Saclay, C2N – Orsay, 91405 Orsay cedex, France; 2 Laboratoire Charles Fabry, Institut d’Optique Graduate School, CNRS, Université Paris Saclay, 2 Avenue Augustin Fresnel, 91127 Palaiseau cedex, France; 3 L-NESS, Dipartimento di Fisica, Politecnico di Milano, Polo di Como, Via Anzani 42, 22100 Como, Italy

## Abstract

Silicon photonics is a large volume and large scale integration platform for applications from long-haul optical telecommunications to intra-chip interconnects. Extension to the mid-IR wavelength range is now largely investigated, mainly driven by absorption spectroscopy applications. Germanium (Ge) is particularly compelling as it has a broad transparency window up to 15 µm and a much higher third-order nonlinear coefficient than silicon which is very promising for the demonstration of efficient non-linear optics based active devices. Si_1−x_Ge_x_ alloys have been recently studied due to their ability to fine-tune the bandgap and refractive index. The material nonlinearities are very sensitive to any modification of the energy bands, so Si_1−x_Ge_x_ alloys are particularly interesting for nonlinear device engineering. We report on the first third order nonlinear experimental characterization of Ge-rich Si_1−x_Ge_x_ waveguides, with Ge concentrations x ranging from 0.7 to 0.9. The characterization performed at 1580 nm is compared with theoretical models and a discussion about the prediction of the nonlinear properties in the mid-IR is introduced. These results will provide helpful insights to assist the design of nonlinear integrated optical based devices in both the near- and mid-IR wavelength ranges.

## Introduction

Chemical and biological sensing devices exploiting the strong rotational-vibrational absorption lines of molecules in the mid-infrared (mid-IR) region of the spectrum, i.e. targeting wavelengths between 3 and 20 µm, are showing a tremendous progress and have been already tested in daily-life applications^1^. In parallel, nonlinear optical phenomena in integrated platforms used for frequency combs, supercontinuum or photon pair generation are subject of intense research with potential applications in molecular spectroscopy^2^, quantum optics^3,4^ and metrology^5^. The extension of the operation wavelength of silicon photonics from the near-IR towards the mid-IR allows taking advantage of the reliable and high-volume fabrication developed in microelectronic foundries to offer higher performance, new functionalities, lower costs, smaller size, reduced weight and low power consumption photonics circuits^6^. The well-known Silicon-on-Insulator (SOI) waveguides have been successfully used to demonstrate nonlinear based optical sources such as frequency combs between 1.5 and 3.3 μm^2^. However, while silicon itself is transparent up to a wavelength of about 8 μm, the SiO_2_-buried layer limits the transmission of SOI waveguides at around 4 μm wavelength. Alternative solutions including silicon-on-sapphire^7^, silicon nitride^8^ or suspended waveguides^9^ have been developed. Among the materials compatible with large volume and large scale integration, germanium (Ge) is particularly compelling as it exhibits a large transparency window from 1.9 to 15 μm^10^. Furthermore, Ge is expected to have a much higher third-order nonlinear coefficient than silicon in mid-IR wavelength range, at which low loss Ge strip waveguides^11,12^, and wavelength (de)multiplexers^13,14^ up to 5.8 μm have been successfully demonstrated. Low loss graded Si_1−x_Ge_x_/Si waveguides were also demonstrated^15,16^ at 4.6 and 7.4 μm wavelengths.

Modeling of the nonlinear optical (NLO) coefficients of the Si_1−x_Ge_x_ alloy as a function of the Ge fraction *x* was performed previously^17^ while the NLO response of Si_1−x_Ge_x_/Si waveguides was experimentally investigated in refs^18–20^, followed by a demonstration of supercontinuum generation in graded Si_1−x_Ge_x_ waveguides on silicon^21^. However, the Ge fraction of Si_1−x_Ge_x_ waveguides was limited to a maximum of 42% in previous experimental studies. An increase of Ge concentration was proposed in ref.^16^, in order to extend the transparency window of Si_1−x_Ge_x_ waveguides but no experimental data related to the NLO coefficients of these Ge-rich Si_1−x_Ge_x_ alloys (*x* > 0.7) are available. In this paper, we report for the first time on the measurements of NLO coefficients of Ge-rich Si_1−x_Ge_x_ waveguides with germanium concentrations ranging from 0.7 to 0.9. A bi-directional top hat D-Scan method^22^ was used to determine the Kerr nonlinear refractive index (*n*_2_) and the two-photon absorption coefficient (${\beta }_{TPA}$) at the wavelength of 1.58 μm serving as a basis for an evaluation of the nonlinear properties of the Si_1−x_Ge_x_ material in the 2–10 μm wavelength range. The experimental values are compared with theoretical predictions and used to discuss the theoretical models. Such an accurate evaluation of the third order nonlinear coefficients in Si_1−x_Ge_x_ alloys will pave the way of forthcoming nonlinear integrated optical designs in a wide range of wavelengths comprising the near- and the mid-IR spectral windows.

### Sample description

In order to experimentally investigate both ${\beta }_{TPA}$ and *n*_2_ coefficients of Ge-rich Si_1−x_Ge_x_ alloys, three different sets of waveguides have been fabricated. They consisted in 2 μm-thick Ge-rich Si_1−x_Ge_x_ waveguide cores with *x* = 0.7, 0.8 and 0.9. These Ge concentrations have been chosen to determine the evolution of the nonlinear parameters around *x* = 0.8, where the minimum bandgap energy changes from being “silicon-like” (E_ΓΔ_) to “germanium-like” (E_ΓL_)^23^. Interestingly, current theoretical models used to estimate the third order nonlinear susceptibility in Si_1−x_Ge_x_ alloys use either an indirect bandgap model for 0 $\le \,x\,\le$ 0.8 or a direct one for 0.8 $< \,x\,\le$ 1^17^, which intrinsically leads to an incongruity for the nonlinear behavior around *x* = 0.8 that makes impossible to predict the material nonlinear properties and furthermore to design specific nonlinear device functionalities nearby that concentration.

**Figure 1:**
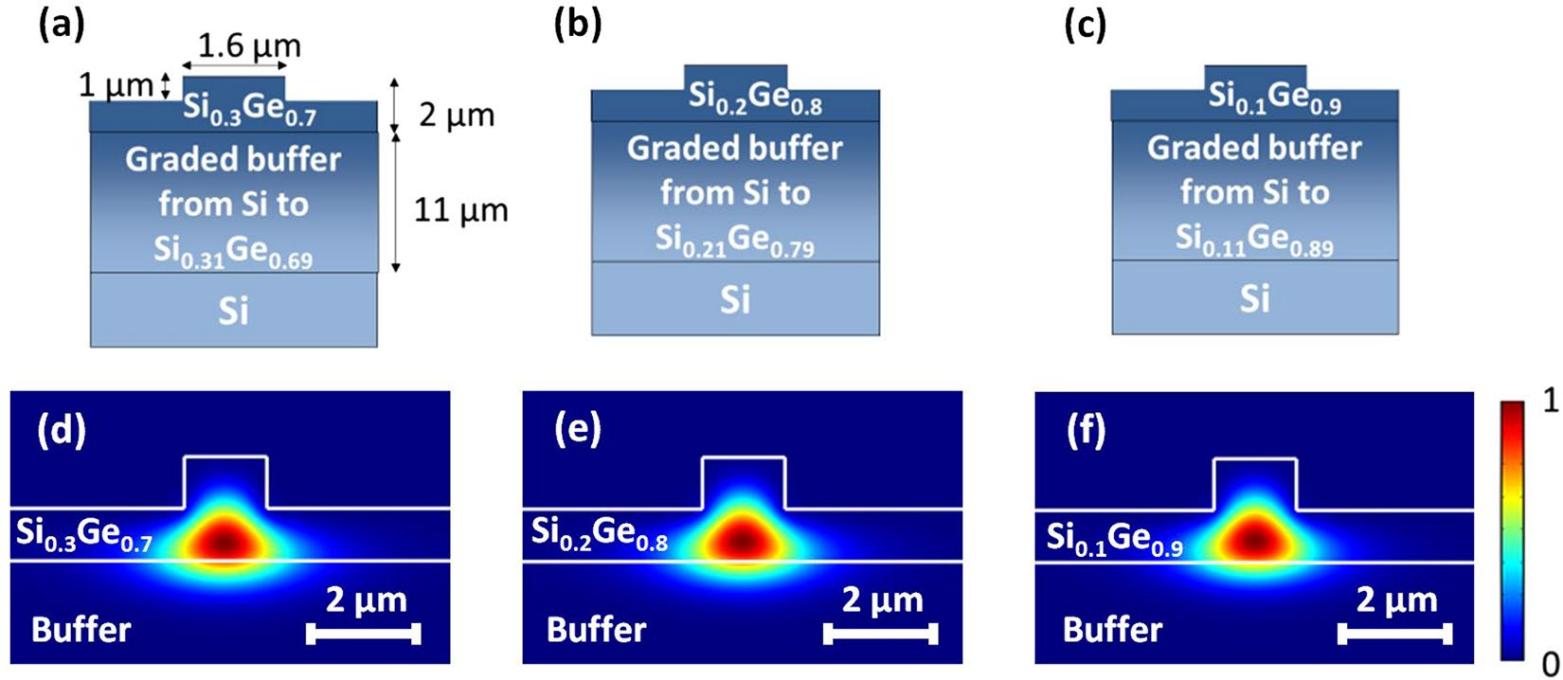
(**a**) to (**c**) Schematic description of the fabricated waveguides. The dimensions are identical for the three devices; (**d**) to (**f**): Normalized ${|\overrightarrow{e}|}^{2}$calculation showing the confinement of the quasi-TE fundamental mode at *λ* = 1.58 μm.

To obtain Ge-rich Si_1−x_Ge_x_ layer grown on silicon with a good quality, an 11 μm-thick graded layer has been used to smoothly accommodate the lattice parameter from Si to the 2 μm-thick Ge-rich Si_1−x_Ge_x_ waveguide, as reported in Fig. 1. This technique is known to provide low threading dislocations in the top layer^24^, while yielding good light confinement thanks to the gradual increase of the refractive index in the graded layer^25^. Low energy plasma enhanced chemical vapor deposition (LEPECVD) has been used for the Si_1−x_Ge_x_ growth. Rib waveguides were then defined to allow lateral mode confinement. Setting the etching depth to 1 μm and the waveguide width to 1.6 μm, as shown in Fig. 1, enables quasi-TE single mode operation at *λ* = 1.58 μm for the three Ge concentrations, namely *x* = 0.7, 0.8 and 0.9. The quasi-TE field modes calculated with Lumerical MODE solutions are shown in Fig. 1(d,e,f) for Ge concentrations of 70%, 80% and 90% respectively. The confinement factor (τ) in the Si_1−x_Ge_x_ top homogeneous region was found to be always larger than 72%. By considering both the top homogeneous region and the top part of the graded buffer where the variation in the germanium concentration is less than 5%, quasi-TE optical mode field confinements reach values above 93%.

The waveguide refractive index maps were modeled by following a linear interpolation between refractive index of Si and Ge at 1.58 µm. The simulated optical mode effective index and nonlinear area of the quasi-TE fundamental mode are reported in Table 1 for the three studied waveguides. The nonlinear area is calculated through the relation ${A}_{NL}=\frac{{(\iint \Re [\overrightarrow{e}\times \overrightarrow{{h}^{\ast }}]\cdot \overrightarrow{z}{d}^{2}\overrightarrow{r})}^{2}}{{\varepsilon }_{0}^{2}{c}^{2}{\iint }_{{s}_{NL}}{n}_{0}^{2}{|\overrightarrow{e}|}^{4}\,{d}^{2}\overrightarrow{r}}$, where $\overrightarrow{e}$ and $\overrightarrow{h}$ refer to the electric and magnetic fields, $\overrightarrow{z}$ a unit vector in the propagation direction, ${\varepsilon }_{0}$ and *c* the permittivity and light velocity in vacuum, *n*_0_ the linear refractive index, and *S*_NL_ the waveguide area. The waveguide lengths are 6 mm, 7 mm and 6 mm respectively for the 0.7, 0.8 and 0.9 Ge concentration waveguides, with linear propagation loss (*α*) measured equal to $2.3\pm 0.5\,{{\rm{cm}}}^{-1},\,2.7\pm 0.6\,{{\rm{cm}}}^{-1},\,4.6\pm 0.9\,{{\rm{cm}}}^{-1}$ by analyzing the device spectral transmissions using a tunable laser source.

**Table 1:** Ge concentration of the waveguide core, simulated effective index and nonlinear area (quasi-TE polarization) of the guided mode at 1.58 μm.

Ge concentration of the waveguide core	Optical mode effective index	${{\boldsymbol{A}}}_{{\boldsymbol{NL}}}\,({\boldsymbol{\mu }}{{\bf{m}}}^{{\bf{2}}})$
70%	4.02	6.44
80%	4.10	5.93
90%	4.18	5.53

### D-Scan nonlinear characterization method

Most of the nonlinear characterization methods rely on output transmission measurements of a pulsed beam injected inside the waveguide. The nonlinear relation between the output power and the injected peak power reveals the presence of multi-photon absorption effects, whereas intensity dependent spectral broadening is related to time varying phase experienced by the pulse. The latter results from the variation of the effective refractive index of the guided mode, which can be induced by free-carrier induced refraction (FCR) variation, thermal or optical Kerr effects. For the purpose of accurately characterizing the nonlinear response of waveguides with sizes smaller than the wavelength, we underline the necessity in systematically determining the coupling efficiency in/out of the photonic integrated circuit. The experimental methods and analysis to quantify these parameters are rarely reported in nonlinear characterizations in the literature.

In this work, we provide nonlinear characterizations that include reliable measurement of the injected power in the waveguide via a bidirectional Dispersive-Scan (D-Scan) method. The D-Scan method, a temporal analogue of the Z-Scan technique^26^, consists in measuring the output spectral broadening of transmitted pulses by varying the dispersion coefficient ${\varphi }^{(2)}$ introduced to the input pulses^27,28^. For Z-Scan, the spatial deformation of a laser beam is analyzed while the nonlinear bulk-material is displaced through the focused spot and for increasing power. The optical (or thermal) Kerr effect induces an intensity dependent spatial variation of the refractive index of the material, playing the role of a spatial lens that modifies the beam propagation. Similarly, the D-Scan technique consists in recording the spectral broadening behaviors experienced by linearly chirped pulses with increasing incident power in order to characterize the temporal Kerr lens introduced by the nonlinear material. There is a complete analogy between Z-Scan and D-Scan as they both rely on the interplay between linear terms, respectively diffraction and dispersion, and nonlinear Kerr effect.

The experimental procedure described below consists in first determining the coupling efficiencies from each waveguide facet and the two photon absorption (TPA) coefficient ${\beta }_{TPA}$ of the guided optical mode via a bidirectional nonlinear transmission experiment. By controlling the chirp coefficient of incident pulses and for various incident power values, the spectral broadening features of the outgoing pulses are analyzed to measure the TPA figure of merit defined as $FO{M}_{TPA}={n}_{2}/({\lambda }_{0}{\beta }_{TPA})$, followed by the nonlinear refractive index *n*_2_ determination, including its sign. By accurately measuring the genuine injected power into the optical guided mode, our method allows to measure the real and imaginary parts of the third order nonlinear contributions of Ge-rich Si_1−x_Ge_x_ waveguides with a 10% precision.

### Experimental set-up and measurements

**Figure 2:**
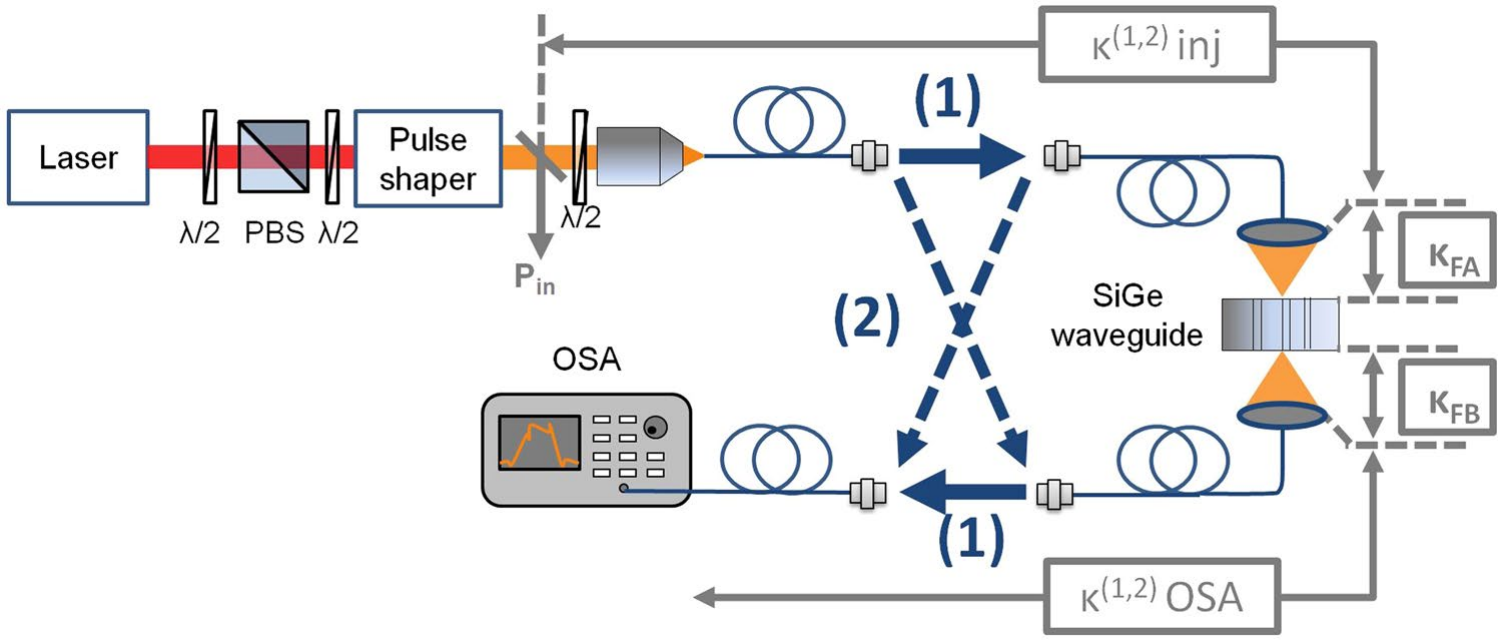
Experimental bidirectional D-Scan set-up. PBS: Polarization Beam Splitter. OSA: Optical Spectrum Analyzer.

The experimental set-up, described in Fig. 2, operates with a mode locked erbium doped fiber laser that delivers 150fs duration pulses, with a repetition rate of $F\,=50\,{\rm{MHz}}$. These pulses are sent through a grating based stretcher that fixes the pulse spectrum following a quasi-rectangular shape of 7.3 nm width and introduces the adjustable dispersion coefficient ${\varphi }^{(2)}$, used to control the spectral phase relation of the pulses before their injection inside the waveguide^29^. For ${\varphi }^{(2)}=\,0\,{{\rm{ps}}}^{2}$, the measured autocorrelation duration is ${T}_{{\rm{autocor}}}\,=\,2\,\,{\rm{ps}}$, which from simulations of a *Sinc*-like temporal pulse shape corresponds to a pulse duration of 1.2 ps. Optical pulses are injected inside the waveguide by means of microscope objective based couplers connected to single mode Polarization-Maintaining (PM) fibres. As shown in Fig. 2, two counter-propagating nonlinear transmissions, (1) and (2), can be performed through a set of fiber connectors. The optical couplers include a half-wave plate followed by a polarization beam splitter (PBS) cube to match with the polarization state of the fundamental TE waveguide mode (not shown in Fig. 2). Note that either a lensed fiber or grating coupler can substitute the microscope objective based coupler used in our set-up. An Optical Spectrum Analyzer (OSA) is then used at the output of the waveguide to measure the optical spectra of the transmitted pulses.

**Figure 3:**
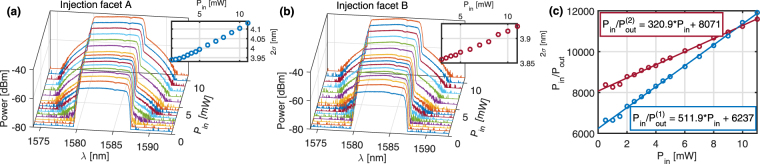
Bi-directional nonlinear transmission characterization of the ${{\rm{Si}}}_{0.1}{{\rm{Ge}}}_{0.9}$ waveguide. (**a**) Output spectra as a function of the input power when injecting from the facet A. (**b**) When injecting from facet B. Inset: spectral r.m.s. linewidth $2\sigma$. (**c**) Bidirectional Pin/Pout vs Pin for the ${{\rm{Si}}}_{0.1}{{\rm{Ge}}}_{0.9}$ waveguide.

For the bidirectional nonlinear transmission measurement, the dispersion coefficient ${\varphi }^{(2)}$ is first set to 0ps^2^, and the transmitted optical spectra are measured for light injected from each sample facets with the incident average power *P*_*in*_ varying from 0.5 to 10 mW. The optical spectra depicted in Fig. 3(a,b) for a ${{\rm{Si}}}_{0.1}{{\rm{Ge}}}_{0.9}$ waveguide exhibit a symmetric broadening, characteristic from a self-phase modulation (SPM) induced by optical Kerr effect that increases with *P*_*in*_. From the calculated spectral r.m.s. linewidth 2*σ*, presented as inset figures, it is noticeable that for ${{\rm{Si}}}_{0.1}{{\rm{Ge}}}_{0.9}$, the SPM nonlinear effects are stronger when injecting from facet A. The common assumption that the input and output coupling coefficients are equal cannot be applied in our case as it would induce a significant error in the nonlinear parameter estimations.

Prior to the analysis of the nonlinear transmission, we recall the relation between the average output power *P*_*out*_ and the average input power *P*_*in*_ in case of a dominant TPA effect^30^:

1 $${P}_{out}=\frac{{\kappa }_{in}{\kappa }_{out}{e}^{-\alpha L}{P}_{in}}{1+\frac{{\beta }_{TPA}}{{A}_{NL}}{\kappa }_{in}\eta {P}_{in}{L}_{eff}}$$

with ${\kappa }_{in}$ and ${\kappa }_{out}$ the input and output coupling coefficients and $\eta$ a parameter defined by ${\rm{\eta }}=1/(F{\int }_{0}^{\frac{1}{F}}{|U(t)|}^{2}dt)$ that takes into account the temporal pulse shape *U*(*t*) and repetition rate *F* of the laser in order to link peak with average powers. Eq. (1) includes the linear propagation losses *α* along the effective interaction length ${L}_{eff}=(1-{e}^{-\alpha L})/\alpha$. This nonlinear relation can be rewritten as

2 $$\frac{{P}_{in}}{{P}_{out}}=\frac{1}{{\kappa }_{in}{\kappa }_{out}{e}^{-\alpha L}}+\frac{{\beta }_{TPA}\eta {L}_{eff}}{{A}_{NL}{\kappa }_{out}{e}^{-\alpha L}}{P}_{in}\,=a+b{P}_{in}$$

where *a* and *b* are measurable parameters which are independent of the injected power. Thus, this equation discloses a linear relation between the ratio ${P}_{in}/{P}_{out}$ and the measured input power *P*_*in*_, with the slope being proportional to the nonlinear coefficient ${\beta }_{TPA}$. From the recorded spectra, the average output power *P*_*out*_ is calculated by integrating each spectrum over all the wavelengths and the variations of ${P}_{in}/{P}_{out}$ with *P*_*in*_ are reported in Fig. 3(c) for the two-injection directions in the ${{\rm{Si}}}_{0.1}{{\rm{Ge}}}_{0.9}$ waveguide. The linear fits plotted with dashed lines in Fig. 3(c) give a set of parameters *a* ^(1)^, *a* ^(2)^, *b* ^(1)^ and *b* ^(2)^ linked to the global input and output coupling losses, ${{\kappa }_{in}}^{(1)}={{\kappa }_{inj}}^{(1)}{\kappa }_{(A)}$, ${{\kappa }_{in}}^{(2)}={{\kappa }_{inj}}^{(2)}{\kappa }_{(B)}$, ${{\kappa }_{out}}^{(1)}={{\kappa }_{OSA}}^{(1)}{\kappa }_{(B)}$ and ${{\kappa }_{out}}^{(2)}={{\kappa }_{OSA}}^{(2)}{\kappa }_{(A)}$, where ${\kappa }_{(A)}$ and ${\kappa }_{(B)}$ stand for the coupling efficiencies of the facets A and B, respectively. ${{\kappa }_{inj}}^{(1)}$, ${{\kappa }_{inj}}^{(2)}$, ${{\kappa }_{OSA}}^{(1)}$ and ${{\kappa }_{OSA}}^{(2)}$ are connection losses identified in Fig. 2 with values measured on the bench. Assuming that the coupling coefficients from each facet are identical in both directions, the coupling losses could be deduced from simple relations that only depend on measured parameters: ${{\kappa }_{(A)}}^{2}={b}^{(1)}/({b}^{(2)}{a}^{(1)}{{\kappa }_{inj}}^{(1)}{{\kappa }_{OSA}}^{(2)}{e}^{-\alpha L})$, and ${{\kappa }_{(B)}}^{2}={b}^{(2)}/({b}^{(1)}{a}^{(2)}{{\kappa }_{inj}}^{(2)}{{\kappa }_{OSA}}^{(1)}{e}^{-\alpha L})$. The coupling efficiencies measured for the 3 tested waveguides with different Ge concentrations are given in Table 2, with uncertainties deduced from the 95% confidence intervals for the fit parameters *a* ^(1)^, *a* ^(2)^, *b* ^(1)^ and *b* ^(2)^. Knowing the coupling efficiencies and using the relations for the coefficients *a* and *b* given in Eq. (2), the nonlinear coefficients ${\beta }_{TPA}$ for the waveguides can be extracted for Ge concentration of 70%, 80% and 90%, which are listed in Table 2. For the uncertainty calculation of ${\beta }_{TPA}$, we have taken into account the uncertainties related to the linear deviation of the curves ${P}_{in}/{P}_{out}$ (i.e. *a* and *b* coefficients), the measured set-up coupling coefficients (${\kappa }_{inj}$ and ${\kappa }_{OSA}$) and to the nonlinear area due to fabrication errors.

**Table 2:** Coupling efficiencies and ${\beta }_{TPA}$ coefficients for the different Ge concentrations deduced from the measurement of the bidirectional nonlinear transmission at 1.58 µm wavelength.

	Coupling efficiency in facet A, ${{\boldsymbol{\kappa }}}_{({\boldsymbol{A}})}$ (**%**)	Coupling efficiency in facet B, ${{\boldsymbol{\kappa }}}_{({\boldsymbol{B}})}$ **(%)**	${{\boldsymbol{\beta }}}_{{\boldsymbol{TPA}}}$ (cm/GW)
${{\rm{Si}}}_{0.3}{{\rm{Ge}}}_{0.7}$	$32\pm 2$	$28\pm 2$	$5.53\pm 0.50$
${{\rm{Si}}}_{0.2}{{\rm{Ge}}}_{0.8}$	$31\pm 2$	$36\pm 2$	$8.08\pm 0.73$
${{\rm{Si}}}_{0.1}{{\rm{Ge}}}_{0.9}$	$36\pm 2$	$19\pm 1$	$18.3\pm 1.7$

**Figure 4:**
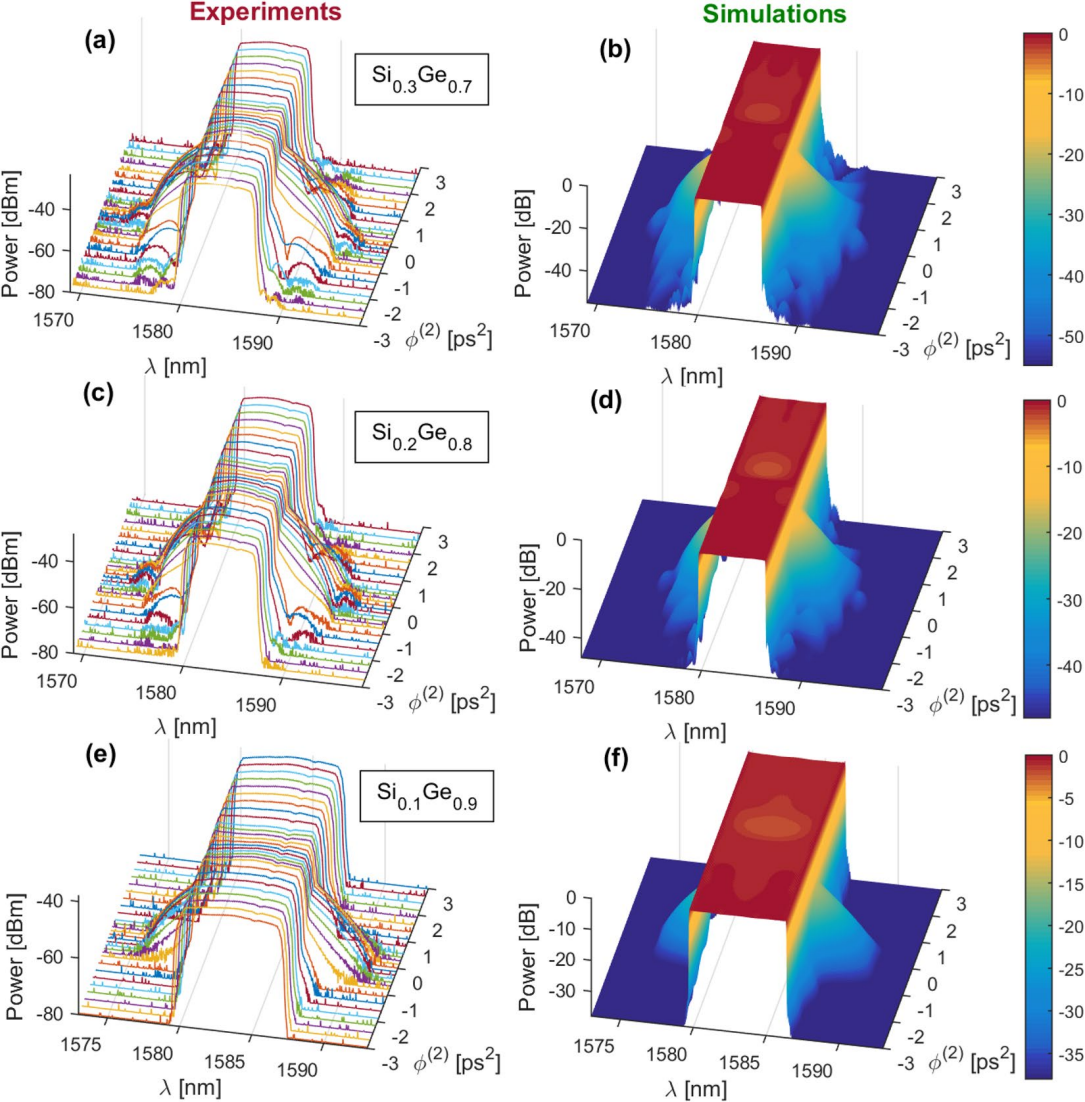
Experimental (figures (**a**,**c**,**e**)) and simulated (figures (**b**,**d**,**f**)) spectra as a function of the second order dispersion for spectral top-hat pulses in the picosecond regime. Figures (**a**) and (**b**) correspond to the ${{\rm{Si}}}_{0.3}{{\rm{Ge}}}_{0.7}$ waveguide, figures (**c**) and (**d**) to the ${{\rm{Si}}}_{0.2}{{\rm{Ge}}}_{0.8}$ waveguide and figures (**e**) and (**f**) to the ${{\rm{Si}}}_{0.1}{{\rm{Ge}}}_{0.9}$ waveguide. The average input power was set to 10 mW.

In order to measure the $FO{M}_{TPA}$ coefficients for each sample, the D-Scan method is applied for which the optical spectra of the output pulses are recorded with dispersion coefficient ${\varphi }^{(2)}$ varying from −3 to $+3\,{{\rm{ps}}}^{2}$, and for input average powers ${P}_{in}=1,\,5,\,7\,$and 10 mW. As an illustration and for ${P}_{in}=10$ mW, the output spectra measured for different ${\varphi }^{(2)}$ are shown in Fig. 4(a,c and e), respectively for the 70%, 80% and 90% Ge-rich Si_1−x_Ge_x_ waveguides. All the initially rectangular shape spectra encounter large spectral variations for ${\varphi }^{(2)}$ comprised between $-1\,{{\rm{ps}}}^{2}$ and $+1\,{{\rm{ps}}}^{2}$ signifying that the pulses undergone a significant SPM effect governed by a nonlinear phase shift ${\varphi }_{NL}$. By reducing the absolute value $|{\varphi }^{(2)}|$, and for a constant incident average power (fixed energy), the injected pulse duration is decreased and the peak intensity is increased, leading the pulse to experience a larger intensity dependent ${\varphi }_{NL}$.

**Figure 5:**
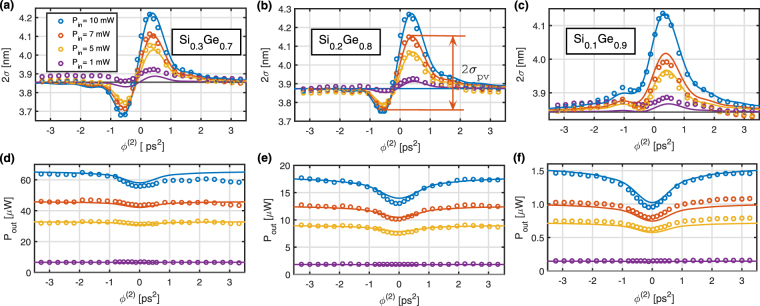
Top Hat D-Scan characterization of the Si_1−x_Ge_x_ waveguides. Figures (**a**) to (**c**) display the measured r.m.s. spectral linewidth as a function of the dispersion coefficients ${\varphi }^{(2)}$ applied to the injected pulses in a 70%, 80% and 90% Ge-rich Si_1−x_Ge_x_ waveguide, respectively, as a function of the input power. Figures (**d**) to (**f**) correspond to the measured output power as a function of ${\varphi }^{(2)}$ for the respective input powers and Ge concentrations.

To analyze the spectral features, the output r.m.s. spectral widths $2{\sigma }_{\lambda }$ are plotted as a function of ${\varphi }^{(2)}$ at various incident powers in Fig. 5(a,b and c), where the circles correspond to the experimental data. The curves present a dispersive shape similar to those reported in Z-Scan or D-Scan, for which the peak-to-valley excursion $2{\sigma }_{PV}$ (identified in Fig. 5(b)) directly informs about the quantity of nonlinear phase shift ${\varphi }_{NL}$ experienced by the pulse^26–28,31^. The maximum spectral broadening is achieved for a positive value of ${\varphi }^{(2)}$, implying that the nonlinear refractive index *n*_2_ for the three Si_1−x_Ge_x_ alloys is positive. Towards large dispersion values, either positive or negative, SPM-induced broadening effect is negligible since the pulse duration is too large to induce nonlinear effects, and the outgoing spectral width tends to that of the input pulse.

In order to determine ${\varphi }_{NL}$ for each graphs plotted in Fig. 5(a,b and c), we have used a semi-analytical simulation to link the peak-to-valley excursion $2{\sigma }_{PV}$ to a ${\varphi }_{NL}$ value. It consists in calculating the chirped pulse envelope at the waveguide output $A(L,t)=\sqrt{I(L,t)}\exp (i{\varphi }_{NL}(L,t))$, where the intensity and the phase are respectively driven by TPA and Kerr effects^30^:

3 $$I(L,t)=\frac{{I}_{0}{|U(t)|}^{2}\exp (-\alpha L)}{1+{\beta }_{TPA}{I}_{0}{|U(t)|}^{2}{L}_{eff}}$$

4 $${\varphi }_{NL}(L,t)=\frac{2\pi }{\lambda }\frac{{n}_{2}}{{\beta }_{TPA}}\,\mathrm{ln}(1+{\beta }_{TPA}{I}_{0}{|U(t)|}^{2}{L}_{eff})$$

with *I*_0_ the injected peak intensity and ${|U(t)|}^{2}$ the temporal shape of the dispersed shape that propagates through the waveguide. The free-carrier effects are neglected, justified by the symmetric spectral broadening reported in Fig. 4. The waveguides dispersion effect is also neglected as the calculated dispersion lengths are much larger than waveguide lengths for the shortest pulse duration ${T}_{0}\approx 1.2\,\mathrm{ps}$ (272 m, 182 m and 108 m for 70%, 80% and 90% Ge concentration respectively). Using Eqs (3) and (4), output spectra are numerically calculated varying the values for the dispersion coefficient ${\varphi }^{(2)}$ and the nonlinear phase shift ${\varphi }_{NL}={\varphi }_{NL}(L,0)$. By calculating the output r.m.s. spectral widths $2{\sigma }_{\lambda }$ for the simulated spectra as a function of ${\varphi }^{(2)}$ for various nonlinear phase shift ${\varphi }_{NL}$, the relation between the peak-to-valley excursion $2{\sigma }_{PV}$ and ${\varphi }_{NL}$ is derived. This relation is shown in ref.^22^ and is used for the present analysis as it only depends on the pulse shape, which is strictly equivalent in both experiences. Using this relation, one is able to retrieve the nonlinear phase shift ${\varphi }_{NL}$ value for each graph depicted in Fig. 5(a,b and c).

**Figure 6:**
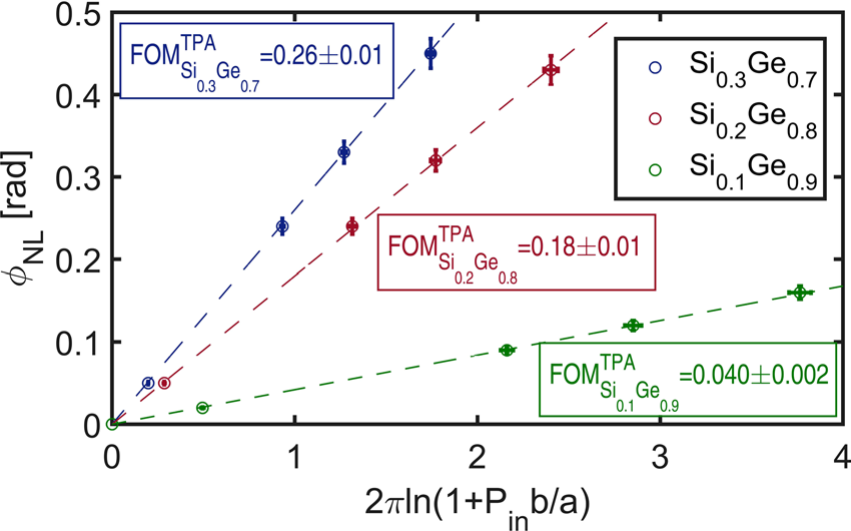
Figure of merit $FO{M}_{TPA}$ measurements for 70%, 80% and 90% Ge-rich Si_1−x_Ge_x_ waveguides deduced from the slope of the linear variation of the nonlinear phase ${\varphi }_{NL}$ with the experimental dependent parameter $2\pi \,\mathrm{ln}(1+\frac{b}{a}{P}_{in})$.

The next step consists in analyzing the dependence of the nonlinear phase shift ${\varphi }_{NL}$ with the incident power to characterize the nonlinear refractive index *n*_2_ of the material. From Eq. (4), the maximum nonlinear phase shift accumulated during the propagation follows the relation:

5 $${\varphi }_{NL}=2\pi FO{M}_{TPA}\,\mathrm{ln}(1+\frac{b}{a}{P}_{in})$$

which depends on the measured parameters *a*, *b*, and ${P}_{in}$. The measured nonlinear phase shifts ${\varphi }_{NL}$ for each sample are then plotted in Fig. 6 as a function of the experimental dependent parameter $2\pi \,\mathrm{ln}(1+\frac{b}{a}{P}_{in})$. The experimental results follow a remarkable linear dependency, in accordance with the expected relation (5). The slopes of the line fits, plotted in Fig. 6 with dotted lines, give a measurement of the $FO{M}_{TPA}$ coefficients for each sample with values listed in Table 3. The uncertainties for $FO{M}_{TPA}$ coincide with the 95% confidence intervals for the line fit slopes, which takes into account the error bars associated to each point of the Fig. 6 plots. The horizontal error bars are deduced from the uncertainties related to the measured parameters *a*, *b*, and ${P}_{in}$. The vertical error bars account for the precision at which the $2{\sigma }_{PV}$ excursions are extracted from the experimental $2{\sigma }_{\lambda }$ curves, and for the simulated relation between $2{\sigma }_{PV}$ and ${\varphi }_{NL}$. The $2{\sigma }_{PV}$ excursions are determined by using a local parabolic fit to measure the maxima and minima of the dispersive curves $2{\sigma }_{\lambda }$. The 95% confidence intervals for the parabolic fit coefficients are used to calculate the uncertainties of the measured $2{\sigma }_{PV}$. Our experimental procedure reaches a noteworthy precision, showing a clear distinction between the three Ge-rich Si_1−x_Ge_x_ alloys for which the $FO{M}_{TPA}$ figure of merits are measured with an accuracy of $\pm 5 \%$. The advantage in using a Top-Hat D-Scan technique, employing rectangular spectral shaped pulses, is revealed by its capability in measuring nonlinear phase shifts as low as 21 mrad for ${P}_{in}=1$ mW (see Fig. 6). Finally, we underline that the $FO{M}_{TPA}$ measurements have been achieved independently of the input and output coupling efficiencies.

**Table 3:** *TPA* figure of merit $FO{M}_{TPA}$ and nonlinear Kerr refractive index (${n}_{2}$) of the different Ge concentration Ge-rich Si_1−x_Ge_x_ waveguides at 1.58 µm measured with the top hat D-Scan technique.

	${\boldsymbol{FO}}{{\boldsymbol{M}}}_{{\boldsymbol{TPA}}}$	${{\boldsymbol{n}}}_{{\bf{2}}}$ ($m{\bf{2}}/{\bf{W}}$)
${{\rm{Si}}}_{0.3}{{\rm{Ge}}}_{0.7}$	$0.26\pm 0.01$	$(22.6\pm 2.3)\times {10}^{-18}$
${{\rm{Si}}}_{0.2}{{\rm{Ge}}}_{0.8}$	$0.18\pm 0.01$	$(23.0\pm 2.3)\times {10}^{-18}$
${{\rm{Si}}}_{0.1}{{\rm{Ge}}}_{0.9}$	$0.040\pm 0.002$	$(11.6\pm 1.1)\times {10}^{-18}$

Using the relation $FO{M}_{TPA}={n}_{2}/({\lambda }_{0}{\beta }_{TPA})$, the measured values for ${\beta }_{TPA}$ coefficients and the $FO{M}_{TPA}$ values, given in Tables 2 and 3 respectively, the nonlinear refractive index values ${n}_{2}$ are calculated. The values are indicated in Table 3 for the 70%, 80% and 90% Ge-rich Si_1−x_Ge_x_ waveguides. Their related uncertainties, deduced from the uncertainty measurements for ${\beta }_{TPA}$ and $FO{M}_{TPA}$, are typically of the order of $\pm 10 \%$.

To confirm the consistency of our results, the experimental output spectra are compared with the simulated spectra that include the measured coefficients. The simulated spectra are displayed in Fig. 4(b,d and f) for different pulse dispersion ${\varphi }^{(2)}$ varying from −3 to + 3 ${{\rm{ps}}}^{2}$, respectively for the three different Ge concentration waveguides. We have set the average input power to its maximum value (${P}_{in}=10\,{\rm{mW}}$) leading to a larger spectral broadening effect. All the simulated spectra are in very good agreement with the measured spectra, reproducing features with 40 dB dynamics. The accordance between experimental and simulated behaviors is particularly relevant in Fig. 5(a,b and c) where the calculated variations of the output r.m.s. spectral widths $2{\sigma }_{\lambda }$ with the dispersion coefficient ${\varphi }^{(2)}$ are plotted with colored lines. Additionally, the average output power variations with ${\varphi }^{(2)}$ are reported in Fig. 5(d,e and f), for input powers ${P}_{in}=1,\,5,\,7\,$and 10 mW, respectively for the three different Ge concentration waveguides. Each graph contains the experimental (open circles) and the simulated (lines) curves, which both exhibit symmetric dips towards ${\varphi }^{(2)}=0\,{{\rm{ps}}}^{2}$, revealing the presence of TPA dependent absorption. The experimental and simulated curves in Fig. 5 show very similar trends. The latter are derived from the theoretical solutions, which include the linear and nonlinear parameters assessed during the experience, and confirm the consistency of our analysis.

## Discussion

Bidirectional nonlinear transmission and top hat D-Scan measurements have been used to characterize the effective nonlinear coefficients (${n}_{2}$ and ${\beta }_{TPA}$) of the optical waveguides fabricated with Ge-rich Si_1−x_Ge_x_ alloys with core concentrations of 70%, 80% and 90%, as reported in Tables 2 and 3. As mentioned in section 1, the overlap factors within the top Si_1−x_Ge_x_ constant composition layer is always larger than 70%, and more than 90% if the optical mode is included in a region with Ge concentration very close to the value of the top Si_1−x_Ge_x_ constant composition layer (variation of less than 5% in the concentration). This allows a comparison of the measurements with the theoretical and previously reported experimental values of Si, Ge and Si-rich Si_1−x_Ge_x_ alloys, for both ${\beta }_{TPA}$ and ${n}_{2}$.

As expected, ${\beta }_{TPA}$ is larger than the values reported in the literature for smaller Ge concentrations^21^. Indeed, up to now, a maximum value of $1.5\,{\rm{cm}}/{\rm{GW}}$ was stated for a ${{\rm{Si}}}_{0.7}{{\rm{Ge}}}_{0.3}$ waveguide at 1550nm^20^, while values ranging from 5.5 to $18\,{\rm{cm}}/{\rm{GW}}$ are reported for Si_1−x_Ge_x_ waveguides with Ge concentration from *x* = 0.7 to 0.9 at similar wavelengths.

Up to now, only theoretical modeling has been discussed in such Ge-rich Si_1−x_Ge_x_ alloys. In ref.^17^, an indirect bandgap model is used for Si-like materials ($0\le x\le 0.8)$ and a direct bandgap model for Ge-like material ($0.8 < x\le 1$). The ${\beta }_{TPA}$ coefficient deduced from this two-bandgap approaches differed by a factor of 100 for Ge concentration around $x=\,0.8$. In order to compare the experimental results with the theoretical predictions^17^, the measured values are reported in Table 4 together with the theoretical values deduced from the direct and indirect bandgap models, respectively.

**Table 4:** Experimental TPA coefficients and theoretical estimations according to ref.^17^ after applying the direct and indirect models from Ge and Si respectively.

	Experimental ${\boldsymbol{\beta }}{\bf{\text{TPA}}}$ (cm/GW)	Direct bandgap model theoretical ${\boldsymbol{\beta }}{\bf{\text{TPA}}}$ (cm/GW)	Indirect bandgap model theoretical ${\boldsymbol{\beta }}{\bf{\text{TPA}}}$ (cm/GW)
${{\rm{Si}}}_{0.3}{{\rm{Ge}}}_{0.7}$	$5.53\pm 0.50$	––	1.51
${{\rm{Si}}}_{0.2}{{\rm{Ge}}}_{0.8}$	$8.08\pm 0.73$	211	1.57
${{\rm{Si}}}_{0.1}{{\rm{Ge}}}_{0.9}$	$18.3\pm 1.7$	550	1.76

Interestingly, none of the direct or indirect bandgap model reproduces the experimental measurements, while the measured ${\beta }_{TPA}$ values are comprised between those obtained by both models. Furthermore, it is noteworthy that for ${{\rm{Si}}}_{0.2}{{\rm{Ge}}}_{0.8}$ and ${{\rm{Si}}}_{0.1}{{\rm{Ge}}}_{0.9}$, both direct / indirect TPA processes could be involved at the wavelength of the experiment (1.58 μm), so diverse contributions could be expected. Indeed, the energy difference between the direct and indirect transition, which is only 0.14 eV for pure Ge, increases up to ≈ 0.6 eV for Si_0.1_Ge_0.9_^32^. As a consequence direct gap transitions, which determine the NLO properties in pure Ge, are much less relevant when only 10% of silicon is added. Actually, the reported values are closer to the indirect bandgap model, with still one order of magnitude difference in the case of $x=0.9$. Our measured ${\beta }_{TPA}$ values, which increases with the Ge content (see Table 4), emphasizes the growing contribution of direct bandgap transitions on top of a non-negligible contribution of indirect bandgap ones.

The reported nonlinear absorption measurements in Ge-rich Si_1−x_Ge_x_ waveguides give the opportunity to perform more precise modeling and understanding of the different mechanisms involved in the direct/indirect processes, as none of the two models adequately predicts the effective TPA response. Although TPA is an unwanted phenomenon for many nonlinear applications, its ultrafast nature can be exploited to realize a large number of all-optical and optoelectronic devices such as switches, modulators, detectors or pulse shapers^33–35^. Giving physical insights on the nonlinear properties of Ge-rich SiGe alloys can improve the modeling of ${\beta }_{TPA}$ as a function of Ge concentration to assist engineering future devices either at telecom or mid-IR wavelengths.

**Figure 7:**
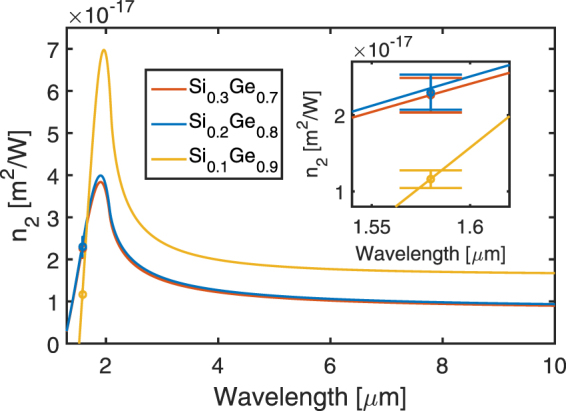
Comparison of theoretical ${n}_{2}$ dispersion curves and experimental measurements for Si_1−x_Ge_x_ alloys with different Ge concentration. The theoretical dispersion curves are based on an indirect bandgap model for x = 0.7 and 0.8, and a direct gap model *x* = 0.9. Inset: zoomed area around the operation wavelength used, at 1.58 µm.

Concerning the experimental Kerr nonlinear refractive index (${n}_{2}$) reported in Table 3, the values do not vary proportionally to the increase of Ge concentration, as is higher for ${{\rm{Si}}}_{0.2}{{\rm{Ge}}}_{0.8}$ than for both ${{\rm{Si}}}_{0.3}{{\rm{Ge}}}_{0.7}$ and ${{\rm{Si}}}_{0.1}{{\rm{Ge}}}_{0.9}$. This result can be explained considering that the measurements are done at a wavelength of *λ* = 1.58 µm, not far from the absorption band edge of the materials. In this region, theoretical modeling predicts strong changes of the ${n}_{2}$ coefficient with possible zero or negative values^17^. A comparison between the measurements and the theoretical modeling is shown in Fig. 7. Following ref.^17^, an indirect bandgap model was applied for $x=\,0.7$ and 0.8, and the multiplicative factor is obtained by fitting the dispersion curves of pure Si with previously reported experiments^36^. For $x=0.9$, the direct bandgap model is used, and the fitting parameter is adjusted by fitting the curve obtained for pure Ge with previously reported experiments^37^. Interestingly, a very good agreement is obtained between the modeling and the experiments for the three considered alloys (${{\rm{Si}}}_{0.3}{{\rm{Ge}}}_{0.7}$, ${{\rm{Si}}}_{0.2}{{\rm{Ge}}}_{0.8}$ and ${{\rm{Si}}}_{0.1}{{\rm{Ge}}}_{0.9}$), which validates the modeling approach for the Kerr nonlinear refractive index. In our operating regime, i.e. around 1580 nm wavelength, ${n}_{2}$ is less sensitive to the details of the band-structure as it was predicted in ref.^17^. Furthermore, we retrieve almost constant values in the midIR that are well comprised between experimental values reported for Si and Ge.

Although complementary experimental data would be required to refine the presented trends specifically in the mid-IR wavelength range, the measured values will be helpful for nonlinear device designers willing to exploit diverse phenomena in different spectral regions. Indeed, Ge and Ge-rich Si_1−x_Ge_x_ alloys have recently become interesting for the implementation of efficient mid-IR non-linear devices^21,38^ from 3 μm, where TPA vanishes, to 15 μm, the transparency limit of Ge. In this wavelength range, a large increase of the nonlinear refractive index of ${{\rm{Si}}}_{1-{\rm{x}}}{{\rm{Ge}}}_{{\rm{x}}}$ alloys has been predicted for $x\, > \,0.8$ ^17^, but there is a lack of experimental confirmation. The presented experimental measurement carried out at *λ* = 1.58 μm, associated with a very good correspondence of the result with the modeling, gives rise to a confident theoretical estimation of ${n}_{2}$ in the mid-IR wavelength range. It is worth noting that an accurate distinction between the different alloys in a strong dispersion regime would not be possible without a precise characterization method. Finally, the values of ${n}_{2}$ ranging between $2.2\times {10}^{-17}$ ${{\rm{m}}}^{2}/{\rm{W}}$ to $1.8\times {10}^{-17}$ ${{\rm{m}}}^{2}/{\rm{W}}$ for ${{\rm{Si}}}_{0.1}{{\rm{Ge}}}_{0.9}$ from 3 to 10 μm wavelength will open the possibility to explore nonlinear phenomena such as four-wave mixing (FWM), continuum generation, and wavelength conversion, which could additionally be integrated with passive and active functionalities by engineering the material bandgap in order to realize complex optoelectronic chips in the long-wave IR domain.

In conclusion, a novel and accurate single beam nonlinear characterization technique was used to report the first measurement of nonlinear third order coefficients in integrated Ge-rich Si_1−x_Ge_x_ alloys with Ge concentrations ranging from *x* = 0.7 to 0.9. As the Ge concentration is raised, an increase of the TPA parameter is observed while the Si_1−x_Ge_x_ Kerr ${n}_{2}$ coefficient does not follow the same monotonous behavior. Counterintuitively, the Kerr coefficient for a concentration of $x=90 \%$ is smaller than for $x=70 \%$ and $x=80 \%$, which can be explained by the energetic proximity between the operation wavelength of the pulsed signal and the bandgap energy of the studied material, where the nonlinearities suffer from strong dispersion. The observed behavior was confirmed by modeling using an indirect bandgap model for ${{\rm{Si}}}_{0.3}{{\rm{Ge}}}_{0.7}$ and ${{\rm{Si}}}_{0.2}{{\rm{Ge}}}_{0.8}$, while a direct gap model was used to simulate the non-linear behavior of ${{\rm{Si}}}_{0.1}{{\rm{Ge}}}_{0.9}$. The global consistency between the reported modeling and experimental results obtained at *λ = *1.58 µm allows an estimation of the expected nonlinearities in the mid-IR wavelength range, where a strong increase of the non-linear refractive index with the Ge concentration larger than $80 \%$ is predicted. Furthermore, we demonstrated that the nonlinear properties of Si_1−x_Ge_x_ waveguides could be tuned through innovative band-gap engineering, giving a comprehensive picture for nonlinear photonics device designers in their efforts to explore these group IV alloys.

## Methods

### Epitaxial growth

Three different epilayers were grown with Ge concentration x in the 2 µm thick guiding core Si_1−x_Ge_x_ of 0.7, 0.8 and 0.9. The epilayers were grown by low energy plasma enhanced chemical vapour deposition (LEPECVD). First, a graded buffer layer Si_1-y_Ge_y_ was deposited at 5–10 nm/s over 11 µm thickness, where y is the linearly increased germanium concentration up to 0.69, 0.79 and 0.89, respectively. It is then followed by the growth of 2 µm thick Si_1−x_Ge_x_ guiding core. Such approach allows having a guiding material of good quality with threading dislocation density (TDD) as low as $3\times {10}^{-6}\,{{\rm{cm}}}^{-2}$. As step of 0.01 in Ge concentration between the termination of the graded buffer and the guiding core provides a step in refractive index which leads to a better confinement of the mode in the guiding core layer.

### Fabrication

Waveguides were patterned with optical lithography followed by an inductively coupled plasma (ICP) etching. The etching depth was fixed to 1 µm for all three epilayers and the waveguide width was fixed to 1.6 µm. A hydrogen peroxide solution (H_2_O_2_) treatment was performed to smoothen the sidewall roughness of the waveguides. Finally, samples were diced for butt coupling experimental setup.

### Nonlinear Optical Characterization

Laser pulses at 50 MHz are sent through a pulse shaper, which introduces a rectangular spectrum and an adjustable 2^nd^ order dispersion coefficient ${\varphi }^{(2)}$. By means of single mode Polarization-Maintaining (PM) fibres connected to microscope objective based couplers, pulses are injected inside the waveguides by a butt-coupling approach. Similarly, the transmitted pulses are injected in PM fibres connected to an Optical Spectrum Analyzer (OSA). The connections between the set of PM fibre patch cables allow achieving two counter-propagating nonlinear transmissions that unveil the modal coupling efficiencies to the waveguide. To quantify the sign and magnitude of the real third order susceptibility, the output r.m.s. spectral width 2σ is analyzed as a function of ${\varphi }^{(2)}$ at various incident powers. The curves allow the measurement of the nonlinear phase shift ${\varphi }_{NL}$induced by optical Kerr effect^22^.

## References

[1] Sieger M, Mizaikoff B (2016). Toward On-Chip Mid-Infrared Sensors. Anal. Chem..

[2] Kuyken B (2015). An octave-spanning mid-infrared frequency comb generated in a silicon nanophotonic wire waveguide. Nat. Commun..

[3] Harris, N. C. *et al*. Integrated Source of Spectrally Filtered Correlated Photons for Large-Scale Quantum Photonic Systems. *Phys*. *Rev*. *X* **4** (2014).

[4] Engin E (2013). Photon pair generation in a silicon micro-ring resonator with reverse bias enhancement. Opt. Express.

[5] Schliesser A, Picqué N, Hänsch TW (2012). Mid-infrared frequency combs. Nat. Photonics.

[6] Zhang, L., Agarwal, A. M., Kimerling, L. C. & Michel, J. Nonlinear Group IV photonics based on silicon and germanium: from near-infrared to mid-infrared. *Nanophotonics* **3** (2014).

[7] Singh N (2015). Midinfrared supercontinuum generation from 2 to 6 μm in a silicon nanowire. Optica.

[8] Foster MA (2011). Silicon-based monolithic optical frequency comb source. Opt. Express.

[9] Soler Penadés J (2014). Suspended SOI waveguide with sub-wavelength grating cladding for mid-infrared. Opt. Lett..

[10] Soref R (2010). Mid-infrared photonics in silicon and germanium. Nat. Photonics.

[11] Chang Y-C, Paeder V, Hvozdara L, Hartmann J-M, Herzig HP (2012). Low-loss germanium strip waveguides on silicon for the mid-infrared. Opt. Lett..

[12] Shen L (2015). Mid-infrared all-optical modulation in low-loss germanium-on-silicon waveguides. Opt. Lett..

[13] Malik A (2013). Germanium-on-silicon planar concave grating wavelength (de)multiplexers in the mid-infrared. Appl. Phys. Lett..

[14] Malik A (2013). Germanium-on-Silicon Mid-Infrared Arrayed Waveguide Grating Multiplexers. IEEE Photonics Technology Letters.

[15] Brun M (2014). Low loss SiGe graded index waveguides for mid-IR applications. Opt. Express.

[16] Ramirez JM (2017). Low-loss Ge-rich Si_0.2_Ge_0.8_ waveguides for mid-infrared photonics. Opt. Lett..

[17] Hon NK, Soref R, Jalali B (2011). The third-order nonlinear optical coefficients of Si, Ge, and Si1- xGex in the midwave and longwave infrared. J. Appl. Phys..

[18] Carletti L (2015). Nonlinear optical response of low loss silicon germanium waveguides in the mid-infrared. Opt. Express.

[19] Carletti L (2015). Mid-infrared nonlinear optical response of Si-Ge waveguides with ultra-short optical pulses. Opt. Express.

[20] Hammani K (2013). Optical properties of silicon germanium waveguides at telecommunication wavelengths. Opt. Express.

[21] Ettabib MA (2015). Broadband telecom to mid-infrared supercontinuum generation in a dispersion-engineered silicon germanium waveguide. Opt. Lett..

[22] Serna SF, Dubreuil N (2017). Bi-directional top hat D-Scan: single beam accurate characterization of nonlinear waveguides. Opt. Lett..

[23] Ferhat M, Zaoui A, Khelifa B, Aourag H (1994). Band structure calculations of GexSi1−x. Solid State Communications.

[24] Isella G (2004). Low-energy plasma-enhanced chemical vapor deposition for strained Si and Ge heterostructures and devices. Solid-State Electron..

[25] Vakarin V (2015). Sharp bends and Mach-Zehnder interferometer based on Ge-rich-SiGe waveguides on SiGe graded buffer. Opt. Express.

[26] Sheik-Bahae M (1990). Sensitive measurement of optical nonlinearities using a single beam. IEEE J. Of Quantum Electron..

[27] Louradour F, Lopez-Lago E, Couderc V, Messager V, Barthelemy A (1999). Dispersive-scan measurement of the fast component of the third-order nonlinearity of bulk materials and waveguides. Opt. Lett..

[28] Fonseca EJS, Gouveia EA, Hickmann JM (2001). Time analogue of the z-scan technique suitable to waveguides. Eur. Phys. J. -At. Mol. Opt. Plasma Phys..

[29] Serna S (2015). Enhanced nonlinear interaction in a microcavity under coherent excitation. Opt. Express.

[30] Yin L, Agrawal GP (2007). Impact of two-photon absorption on self-phase modulation in silicon waveguides. Opt. Lett..

[31] Zhao W, Palffy-Muhoray P (1993). Z-scan technique using top-hat beams. Appl. Phys. Lett..

[32] Xu C, Gallagher JD, Senaratne CL, Menéndez J, Kouvetakis J (2016). Optical properties of Ge-rich Ge1−x Six alloys: Compositional dependence of the lowest direct and indirect gaps. Physical Review B.

[33] Sang X, Tien E-K, Boyraz O (2009). Applications of two photon absorption in silicon. J. Optoelectron. Adv. Mater..

[34] Liang TK (2002). Silicon waveguide two-photon absorption detector at 1.5 μm wavelength for autocorrelation measurements. Appl. Phys. Lett..

[35] Shen L (2015). Two-photon absorption and all-optical modulation in germanium-on-silicon waveguides for the mid-infrared. Opt. Lett..

[36] Wang T (2013). Multi-photon absorption and third-order nonlinearity in silicon at mid-infrared wavelengths. Opt. Express.

[37] Wynne JJ (1969). Optical third-order mixing in GaAs, Ge, Si, and InAs. Phys. Rev..

[38] Ramirez JM (2017). Ge-rich graded-index Si1-xGex waveguides with broadband tight mode confinement and flat anomalous dispersion for nonlinear mid-infrared photonics. Opt. Express.

